# Comparison of iron-reduced and iron-supplemented semisynthetic diets in T cell transfer colitis

**DOI:** 10.1371/journal.pone.0218332

**Published:** 2019-07-05

**Authors:** Anamarija Markota, Rebecca Metzger, Alexander F. Heiseke, Lisa Jandl, Ezgi Dursun, Katharina Eisenächer, Wolfgang Reindl, Dirk Haller, Anne B. Krug

**Affiliations:** 1 Institute for Immunology, Biomedical Center, Ludwig-Maximilians-University Munich, Martinsried, Germany; 2 Chair for Nutrition and Immunology, Technical University Munich, Freising, Germany; 3 Klinikum Mannheim, II. Medizinische Klinik, Mannheim, Germany; Duke University, UNITED STATES

## Abstract

Clinical observations in inflammatory bowel disease patients and experimental studies in rodents suggest that iron in the intestinal lumen derived from iron-rich food or oral iron supplementation could exacerbate inflammation and that iron depletion from the diet could be protective. To test the hypothesis that dietary iron reduction is protective against colitis development, the impact of iron reduction in the diet below 10 mg/kg on the course of CD4+ CD62L+ T cell transfer colitis was investigated in adult C57BL/6 mice. Weight loss as well as clinical and histological signs of inflammation were comparable between mice pretreated with semisynthetic diets with either < 10mg/kg iron content or supplemented with 180 mg/kg iron in the form of ferrous sulfate or hemin. Accumulation and activation of Ly6C^high^ monocytes, changes in dendritic cell subset composition and induction of proinflammatory Th1/Th17 cells in the inflamed colon were not affected by the iron content of the diets. Thus, dietary iron reduction did not protect adult mice against severe intestinal inflammation in T cell transfer induced colitis.

## Introduction

Inflammatory bowel diseases (IBD)—ulcerative colitis and Crohn’s disease—are chronic inflammatory disorders of the gastrointestinal tract resulting from a dysregulated immune response to the intestinal microbiota which is influenced by the genetic susceptibility of the host and environmental factors [1]. In addition to dietary intake of macronutrients (fat, carbohydrates and proteins), micronutrients including iron influence the epithelial barrier function, the mucosal immune response and directly or indirectly the microbiota [2]. The consumption of red meat containing heme iron has been associated with a higher risk for IBD and colorectal cancer [3, 4]. Oral iron supplementation is often avoided during phases of active IBD, because it is poorly tolerated by some IBD patients and may promote IBD symptoms [5]. Results of recent clinical studies however do not provide clear evidence for exacerbation of IBD as a consequence of oral iron supplementation [6]. Animal studies performed in rodents using chemically induced erosive colitis models demonstrated a proinflammatory effect of iron in the intestinal lumen by showing that high dose oral iron supplementation causes an increase in disease activity, inflammatory score and oxidative stress [7–12]. Luminal iron, especially in the form of heme iron was shown to induce oxidative stress and cytotoxicity in the intestinal epithelium [13]. However, it was shown recently that exposure of intestinal macrophages to hemin inhibited their expression of LPS-induced proinflammatory cytokines and this effect was reversed by dietary iron reduction [14]. The effect of dietary iron reduction for the development of intestinal inflammation has been explored in the spontaneous Crohn’s disease-like ileitis model in tumor necrosis factor (TNF)^ΔARE^ mice, which developed less severe intestinal inflammation when treated with an iron-reduced diet [15]. In this study, mice fed with an iron-free (< 10 mg/kg) semisynthetic diet for 11 weeks depleted hepatic iron stores without developing anemia and the protective effect of the iron-reduced diet was still observed when systemic iron stores were repleted by parenteral iron administration demonstrating that luminal iron depletion was responsible for the observed effect [15]. However, iron reduction has not been tested as dietary intervention in the T cell transfer induced colitis model, which causes T cell driven microbiota-dependent colonic inflammation resembling human IBD.

How luminal iron could promote intestinal inflammation is not fully understood. Excess luminal iron induces reactive oxygen species (ROS) and nitric oxide (NO) production which activate the nuclear factor (NF)-κB signalling pathway and induce inflammatory cytokine production [8, 9, 13, 16]. Luminal iron was also shown to trigger endoplasmic reticulum (ER)-stress leading to apoptosis of intestinal epithelial cells and alterations in the composition of the intestinal microbiota [15], but little is known about how changes in the concentration of luminal iron affect intestinal immune responses. Especially the impact of dietary iron on the mononuclear phagocyte (MNP) system and potential subsequent effects on T cell responses during colitis has not been investigated.

The MNP system in the intestine is composed of macrophages and dendritic cells (DCs) that have complementary yet distinct functions [17]. Intestinal CX3CR1^high^ macrophages are equipped with transepithelial dendrite extensions that enable them to sense and internalize microbial antigens and micronutrients (including iron) in the gut lumen [18, 19]. Macrophages are equipped to take up, store and export iron, but may accumulate iron under inflammatory conditions [20]. Iron overload triggers an unrestrained M1 type proinflammatory program in macrophages as observed for example in chronic venous leg ulcers and atherosclerosis [21, 22]. Resident anti-inflammatory Ly6C^low^ intestinal macrophages [23, 24] are continuously replenished by Ly6C^high^ blood-derived monocytes [25] that then downregulate Ly6C and upregulate MHCII, CD64 and F4/80 [26]. During inflammation, Ly6C^high^ monocytes are massively recruited to the colon and differentiate into proinflammatory cytokine producing macrophages which promote and maintain pathogenic T helper (Th) cells producing IL-17 (Th17) and Interferon (IFN)-xγ (Th1) in the inflamed colon [27]. Luminal antigens taken up by non-migratory macrophages can be transferred to intestinal DCs which migrate to the draining lymph nodes (LN) where they present antigens and induce T helper (Th) cell polarization into regulatory (Treg) and effector Th cells [17, 28]. The CD103+ CD11b- intestinal DCs which preferentially induce gut-homing Tregs were found to be decreased in the lamina propria of the colon during colitis in mice and humans concomitant with an increase in the percentage of CD11b+ DCs, which promote differentiation of proinflammatory effector Th cells [29, 30, 31, 32].

Here we investigated the impact of dietary iron reduction in T cell transfer induced murine colitis as a model of human IBD focusing on the effect of luminal iron on intestinal macrophages and DCs as well as Th cell differentiation. Colitis activity was comparable in mice pretreated with iron-reduced and iron-supplemented semisynthetic diets in T cell transfer colitis and no significant differences in the macrophage and DC compartment or in the proinflammatory Th1/Th17 cell frequency in the inflamed colon were observed. Thus, our results show that dietary iron reduction neither changed the intestinal inflammatory immune response nor altered the clinical course of murine experimental colitis induced by T cell transfer.

## Methods

### Mice

Wild type and *Rag1*^*-/-*^ mice (C57BL/6) were bred and held in the mouse facility of the Institute of Medical Microbiology, Immunology and Hygiene at the Technical University Munich. Health monitoring was performed according to the recommendations of the Federation of European Laboratory Animal Science Association (FELASA). Sentinels occasionally tested positive for *Helicobacter* spp. Mice entered experiments at 10–12 weeks of age. Mice were sacrificed by cervical dislocation. All experimental procedures involving mice were performed in accordance with the regulations of and were approved by the local government (Regierung von Oberbayern, Az 205–2012).

### Dietary treatment

The following diets were used: iron-deficient experimental diet (Altromin C1038, <10 mg Fe/kg, Altromin, Lage, Germany), iron sulphate containing experimental diet (FeSO_4_, Altromin C1000, 180 mg Fe/kg) or iron-deficient experimental diet (Altromin C1038) supplemented with hemin (Sigma Aldrich, Seelze, Germany), which was incorporated into the diet (equalling 180 mg Fe/kg). Mice were held on standard chow (Harlan Global rodent diet 2018, Harlan, Germany) until the age of 10–12 weeks, which was then gradually replaced by the experimental diets over a period of 2 weeks. Mice were fed experimental diets for 9 weeks and were then sacrificed for analysis or subjected to T cell transfer colitis and continued on the experimental diets until the end of the experiments. Mice were held in small groups of 3–4 mice per cage throughout the entire experimental time.

### Colitis model

For T cell transfer colitis, 3 x 10^5^ CD4^+^CD62L^+^ splenic T cells were injected intraperitoneally into *Rag1*^*-/-*^ mice. Colitis activity was evaluated by bodyweight measurements and cumulative clinical score (0: absent symptoms, 1: mild symptoms, 2: moderate, 3: severe for inactivity, hunched posture, ruffled fur, diarrhea, rectal prolapse and rectal bleeding). Mice were sacrificed when they had lost up to 20% bodyweight compared to the starting weight or when clinical criteria for euthanasia were reached.

### T cell isolation

For T cell transfer, T cells were isolated from splenocytes of C57BL/6 WT mice (fed normal chow) using CD4^+^ CD62L^+^ Isolation Kit II (Miltenyi Biotech, Bergisch-Gladbach, Germany). The purity of CD4^+^ CD62L^+^ T cells as well as the percentage of Foxp3^+^ Tregs within the isolated T cell population was evaluated by FACS analysis before each transfer experiment (purity: > 90% CD4^+^ CD62L^+^ containing 7–10% Foxp3^+^ as shown previously [33]).

### Isolation of cells from mLNs and colon

Mesenteric lymph nodes (mLNs) were minced and digested with collagenase D (500 μg/mL) and DNase I (100 μg/mL) (Roche, Mannheim, Germany) at 37°C for 30 min. The digested tissue fragments were passed through a 100 μm cell strainer.

Colons were excised and attached mesenteric fat was carefully removed. The whole colons were cut longitudinally and flushed 3 times with ice cold Ca^2+^/Mg^2+^ free HBSS (Invitrogen, Thermo Fisher Scientific, Waltham, MA, USA) and then cut in 3 mm long pieces and incubated in Ca^2+^/Mg^2+^-free HBSS with 2 mM DTT (Roth, Karlsruhe, Germany) and 5 mM EDTA (Invitrogen, Thermo Fisher Scientific) for 30 min at 37°C stirring in the incubator (5% CO_2_). The treated colon pieces were collected using a 100 μm filter. The filtrate was further passed through glass wool (Roth) to remove epithelial cells and isolate the IELs. For isolation of LPLs, the remaining colon pieces were digested in RPMI 1640 medium (Thermo Fisher Scientific) containing DNase I (100 μg/mL), collagenase D (500 μg/mL) and collagenase V (850 μg/mL, Sigma Aldrich, St. Louis, MO, USA) for 30 min at 37°C and passed through a 100 μm cell strainer.

### Flow cytometry

Cells were stained with fluorescently labelled antibodies against the following surface markers: CD3ε CD4, CD11b, CD11c, CD45.2, I-A^b^ (all eBioscience, San Diego, CA, USA), CD64 (BioLegend, San Diego, CA, USA), CD62L, CD103, Ly6-C (BD Biosciences, Heidelberg, Germany) at 1:200 final dilution in FACS buffer (PBS, 2% FCS) with 50% HB197 hybridoma supernatant containing Fc blocking antibody 2.4G2 (ATCC, Manassas, VA, USA). Dead cells were excluded by propidium-iodide (PI) staining (Sigma Aldrich). For intracellular cytokine staining single cells from mLNs and colonic LPLs were resuspended in complete medium (RPMI 1640, 1% GlutaMAX-I, 1% non-essential amino acids, 1% Penicillin/Streptomycin, 1 nM Sodium Pyruvate Solution (all from Invitrogen) and β-mercaptoethanol (Sigma-Aldrich), Cell were stimulated at 10^6^ cells/96-well with 20 ng/mL Phorbol 12-myristate 13-acetate (PMA) (Sigma-Aldrich), 1 μg/ml Ionomycin (Sigma-Aldrich), Golgi Plug (0.2% v/v) and Golgi Stop (0.14% v/v) (BD Biosciences) for 6h at 37°C. Cells were stained for CD3ε and CD4 and then treated with IC fixation buffer and 1X Permeabilization Buffer (eBioscience). Intracellular staining was performed using anti IL-17A-APC and anti IFN-γ-PE antibodies (BD Bioscience). Intracellular Foxp3 staining was performed using the Intracellular Foxp3 staining kit (eBioscience). Flow cytometry analysis was performed using a Gallios Flow cytometer (Beckman Coulter, Brea, CA, USA) and data were analysed using FlowJo software (Tree Star, Stanford, USA).

### Histology

Colons were cut longitudinally and “swiss rolls” were prepared and fixed in 4% formaldehyde (Roti-Histofix, Karl-Roth, Karlsruhe, Germany) over night and embedded in paraffin. Tissue blocks were cut in 4 μm sections and stained with hematoxylin and eosin (H&E). Histological scoring was performed observer-blinded by adding the scores (0–3) for leucocytes infiltration and epithelial damage as described (maximal score 6) [34].

### Statistical analysis

Statistical analysis was performed using Graph Pad Prism software (Version 6.0, GraphPad Software San Diego, CA, USA). The results are presented as mean ± SD. One way ANOVA test followed by Tukey`s post-hoc test was used for multiple comparisons and histology scores were compared using Kruskal-Wallis non-parametric test. p values < 0.05 were considered to indicate significant differences.

## Results

### Dietary iron reduction does not protect against colitis induced by CD4+ CD62L+ T cell transfer in Rag1-/- mice

**Fig 1:**
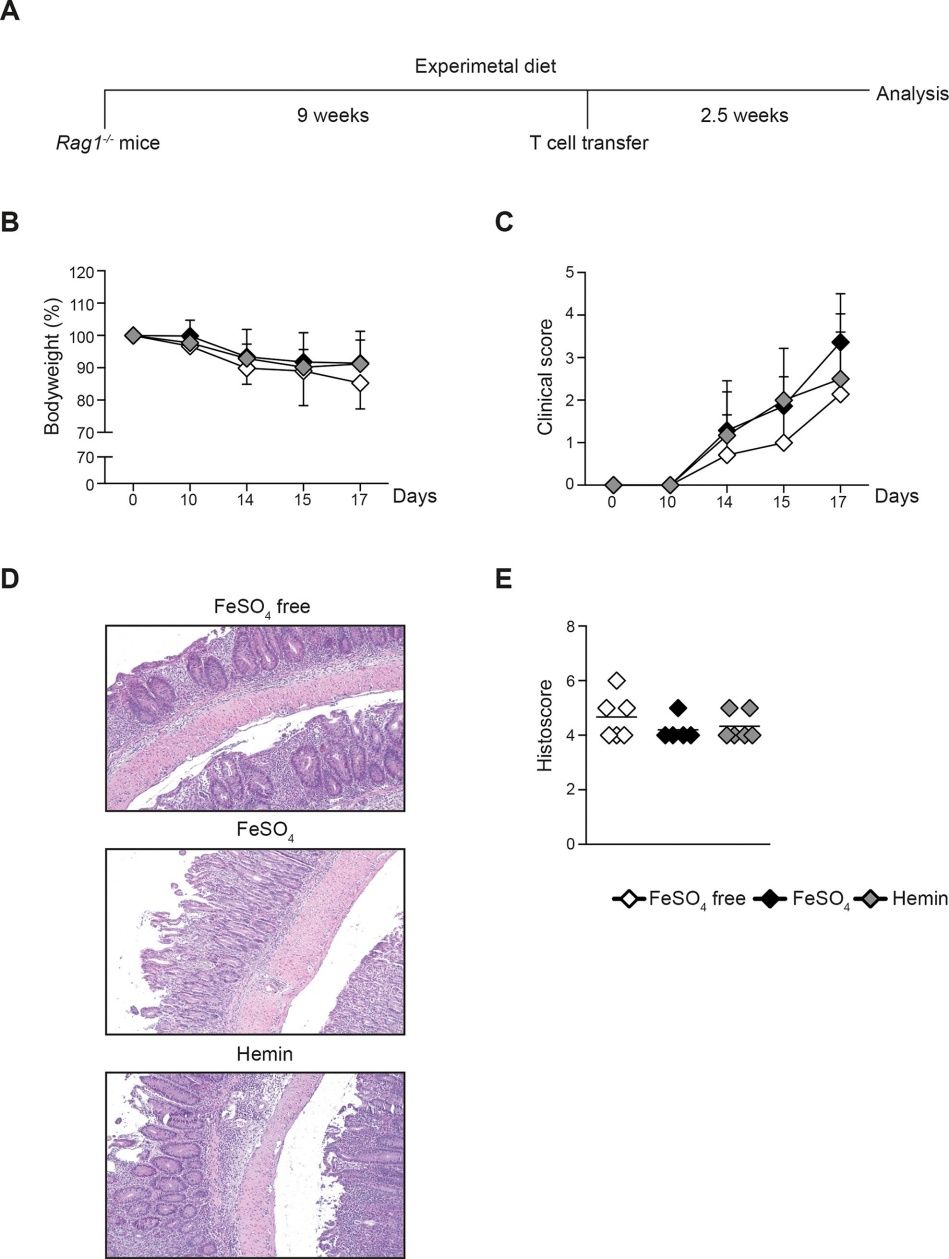
Dietary iron reduction does not protect against T cell transfer colitis. **(A)** Experimental design for T cell transfer colitis in *Rag1*^*-/-*^ recipient mice fed with iron depleted (w/o Fe) or iron supplemented experimental diets (FeSO_4_ or Hemin). **(B)** Bodyweight development at indicated time points after transfer of CD4^+^ CD62L^+^ T cells to *Rag1*^*-/-*^ mice fed with experimental diets (one of 3 independent experiments with 4–6 animals in each group is displayed; mean ± SD; one way ANOVA, not significant). **(C)** Clinical colitis scores at indicated time points of mice from (A) (one of 3 independent experiments with 4–6 animals in each group is displayed; mean ± SD; one way ANOVA, n.s.). **(D)** Representative H&E staining of colon tissues sections of mice from (A) at day 17 after T cell transfer. **(E)** Histological colitis scores of mice from (A) (each symbol represents one animal and horizontal lines indicate mean values for each dietary group; Kruskall-Wallis test, n.s.).

It was shown previously that oral iron supplementation influences chemically-induced colitis activity in rodents and that dietary iron reduction below 10 mg/kg protects mice from spontaneous Crohn’s disease-like ileitis in tumor necrosis factor (TNF)^ΔARE^ mice when compared to normal dietary iron content. Therefore, our hypothesis was that dietary iron reduction below 10 mg/kg could protect mice from colitis and this was tested in C57BL/6/Rag1^-/-^ mice using the CD4^+^ CD62L^+^ T cell transfer colitis model (see experiment outline in Fig 1A). Hemin was used to mimic heme iron contained in red meat or in hemoglobin from mucosal bleeding for example. Mice on iron-depleted diet did not develop anemia as determined by blood hemoglobin concentration ≥ 13.9 g/dl [35] (w/o Fe: 14.9 ± 3.9; FeSO_4_: 16.4 ± 3.6; Hemin: 15.8 ± 3.4 g/dl; table A in S1 File). After T cell transfer mice lost weight progressively in all 3 groups and by day 17 more than 50% of the mice in each group had lost 15–20% of their starting bodyweight (w/o Fe: 85.3 ± 8.0, FeSO_4_: 91.4 ± 9.9, Hemin; 91.2 ± 7.4, mean ± SD, Fig 1B). The bodyweight loss was accompanied by similarly increased clinical disease activity in all experimental groups (clinical score: w/o Fe: 2.1 ± 1.9, FeSO_4_: 3.4 ± 1.1, Hemin: 2.5 ± 1.1, mean ± SD, Fig 1C). Leucocytosis and increased haematocrit (indicating fluid loss) were observed in all dietary groups during colitis (table A in S1 File). The histological colitis score was also comparable between the 3 groups (w/o Fe: 4.7 ± 0.8, FeSO_4_: 4.2 ± 0.4, Hemin: 4.3 ± 0.5, Fig 1D). Similar degrees of epithelial damage with presence of transmural ulcerations and extensive immune cell infiltration were observed in the colon sections (Fig 1E). Ferric iron was detected by histochemical staining in the lamina propria of the inflamed colon at a higher level in mice which had received iron-supplemented diets than in mice which were iron-depleted diet (Fig A in S1 File). These results show that the induction and development of T cell transfer colitis was not affected by reduction of the iron concentration in the diet or by the form of iron (ferrous iron sulphate or hemin), which was added to the experimental diet.

### Recruitment of Ly6C^high^ monocytes to the colon is not impaired by dietary iron reduction during T cell transfer colitis

**Fig 2:**
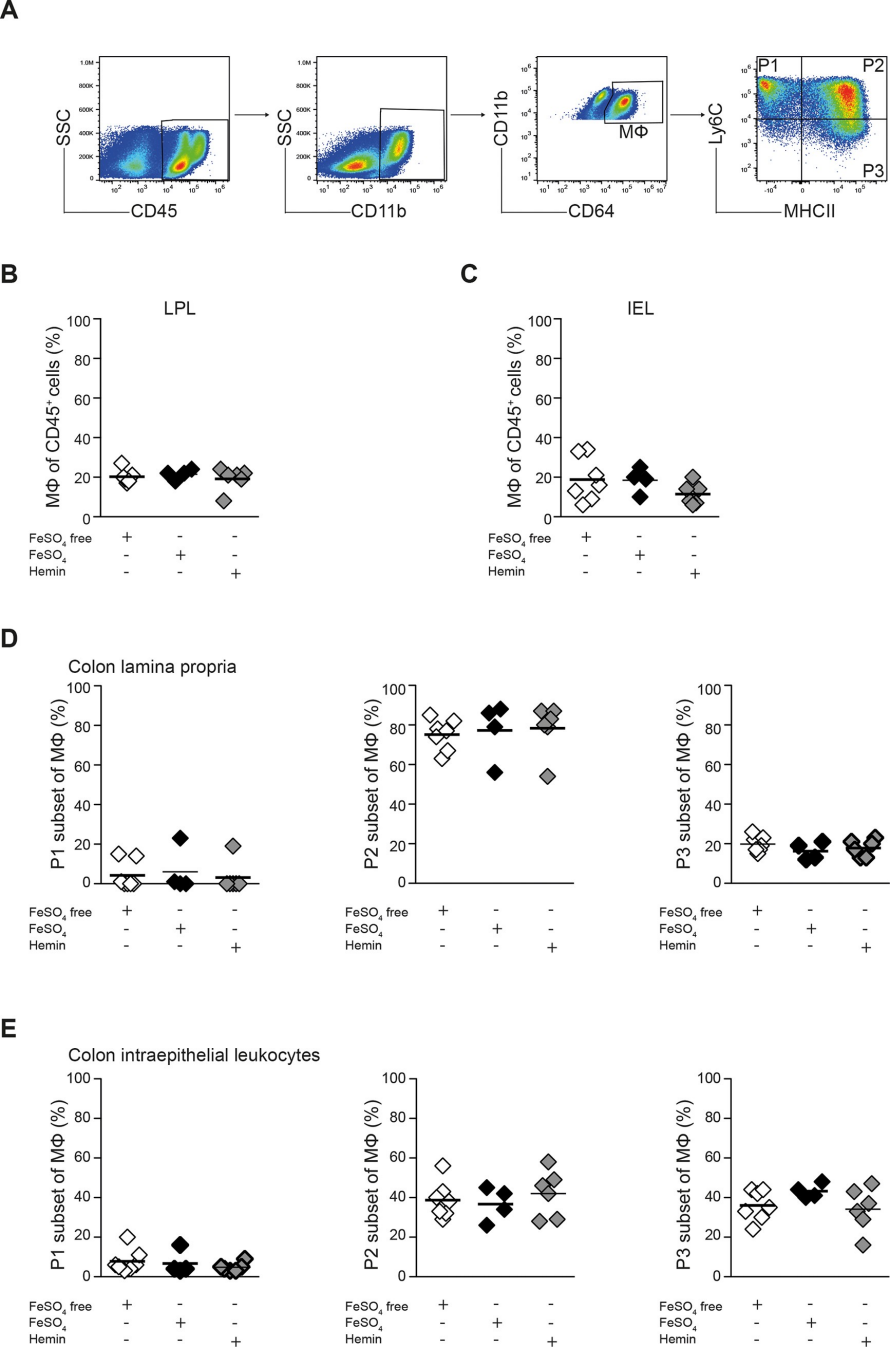
No effect of luminal iron depletion on monocyte/macrophage composition in the inflamed colon. *Rag1*^*-/-*^ mice were treated with iron depleted (w/o Fe) or iron supplemented experimental diets (FeSO_4_ or Hemin) and colitis was induced by T cell transfer. Colon LPLs and IELs were prepared on day 17 after T cell transfer and analyzed by flow cytometry. **(A)** Gating strategy used for characterization of the monocyte/macrophage compartment in LPL and IEL colon fraction. **(B, C)** The percentages of CD11b^+^ CD64^+^ monocytes/macrophages within viable CD45^+^ leucocytes in LPL (B) and IEL (C) colon fractions are shown. **(D, E)** The percentages of P1, P2 and P3 subsets in LPL (D) and IEL (E) colon fractions are shown. (B-E) Results of one representative of 2 independent experiments with 4–6 animals in each dietary group are shown. Crossbars indicate mean values; one way ANOVA, n.s.).

The composition of colonic MNPs changes dramatically during colitis and the increased influx of blood-derived monocytes into the colon is a sensitive parameter of inflammation. To investigate whether the luminal iron content has an impact on monocyte recruitment and maturation, the monocyte/macrophage compartment in the colon was analysed by flow cytometry after exposure to the experimental diets during T cell transfer colitis (Fig 2A). The frequencies of CD11b^+^CD64^+^ macrophages in colon lamina propria leucocyte (LPL) and intraepithelial leucocyte (IEL) fractions were comparable between the groups (Fig 2B and 2C). Analysis of Ly6C and MHCII expression in this population distinguished three distinct subsets: Ly6C^high^MHCII^-^ (P1) and Ly6C^high^MHCII^+^ (P2) cells are derived from circulating Ly6C^high^ monocytes and give rise to Ly6C^low^MHCII^+^ macrophages (P3) [25, 26]. The composition of the monocyte/macrophage compartment in the colon was not affected by changes in dietary iron content as reflected in similar frequencies of P1, P2 and P3 subsets in the 3 experimental groups during colitis (Fig 2D and 2E).

**Fig 3:**
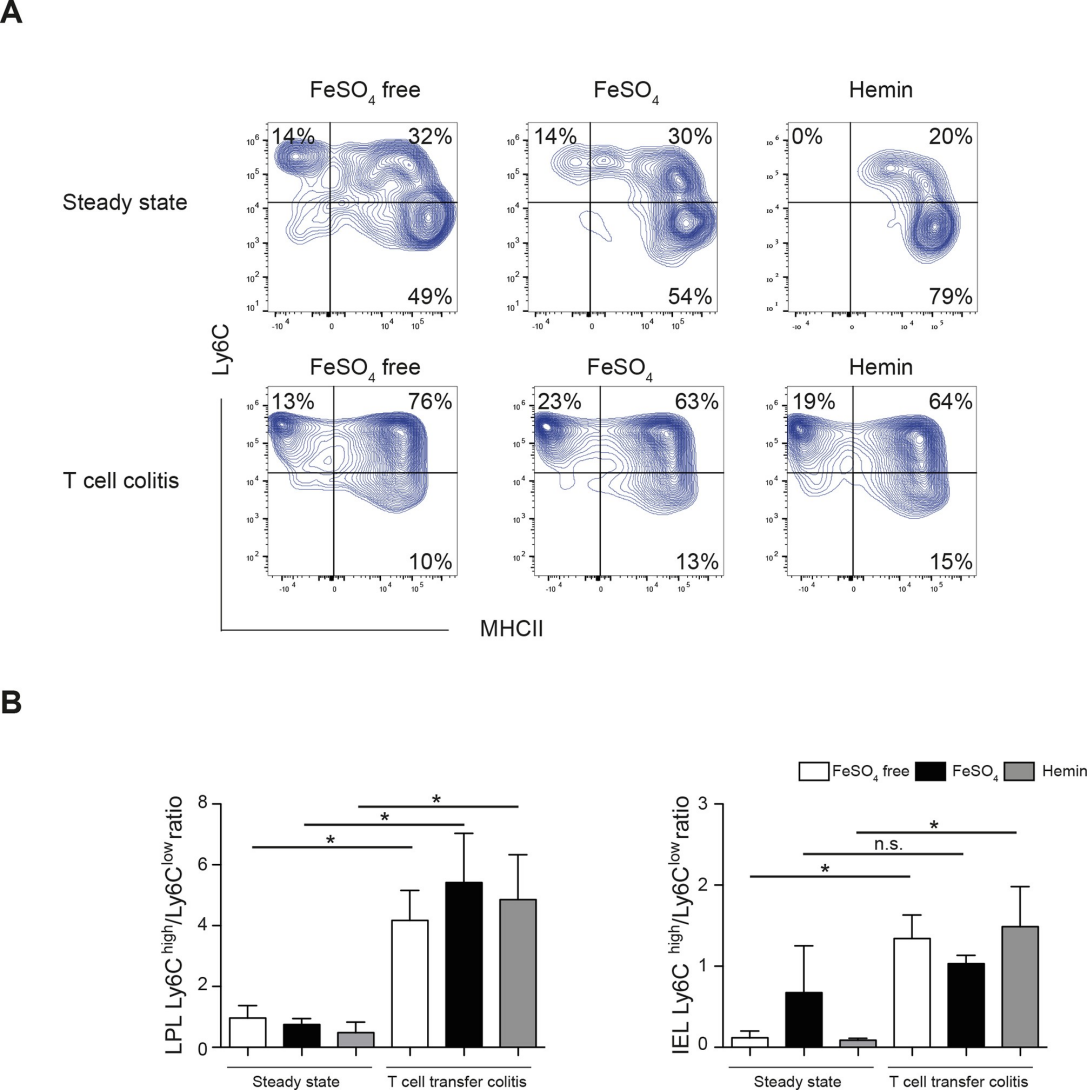
Recruitment of monocytes to the colon during colitis is not influenced by dietary iron reduction. **(A)** The expression of Ly6C and MHCII by CD11b^+^ CD64^+^ colonic monocyte/macrophages in the steady state and on day 17 of T cell transfer colitis in *Rag1*^*-/-*^ mice fed with experimental diets is shown in the dot plots. Numbers in the quadrants indicate percentages. Representative results of one of 2 independent experiments are shown. **(B)** Ratios of Ly6C^high^(P1/P2): Ly6C^low^(P3) CD11b^+^ CD64^+^ cells in colon LPL and IEL isolated from *Rag1*^*-/-*^ mice fed with experimental diets without colitis induction and on day 17 after colitis induction by T cell transfer. An increase in the Ly6C^high^ to Ly6C^low^ ratio indicates recruitment of monocytes to the inflamed colon. Representative results of one of 2 independent experiments with 4–6 animals per each dietary group are shown (mean ± SD; one-way ANOVA with Tukey’s multiple comparison test, n.s.).

The development of colitis after T cell transfer resulted in a marked shift of the Ly6C^low^ P3 population to the Ly6C^high^ MHCII^+/-^ population (P1/P2) in colon LPL compared to *Rag1*^*-/-*^ mice treated with the same type of the diet without T cell transfer in all conditions. The ratio of Ly6C^high^ (P1/P2) to Ly6C^low^ (P3) populations increased during colitis to a similar extent in all 3 experimental groups and was not affected by the dietary treatment itself (Fig 3A and 3B). These results indicate that neither the constant migration of monocytes into the colon and their maturation into Ly6C^low^MHCII^+^ macrophages in the steady state nor the increased influx of blood-derived monocytes into the colon during colitis were affected by modulation of dietary iron in the T cell transfer colitis model.

### Intestinal DC subset composition is not affected by dietary iron reduction during T cell transfer colitis

**Fig 4:**
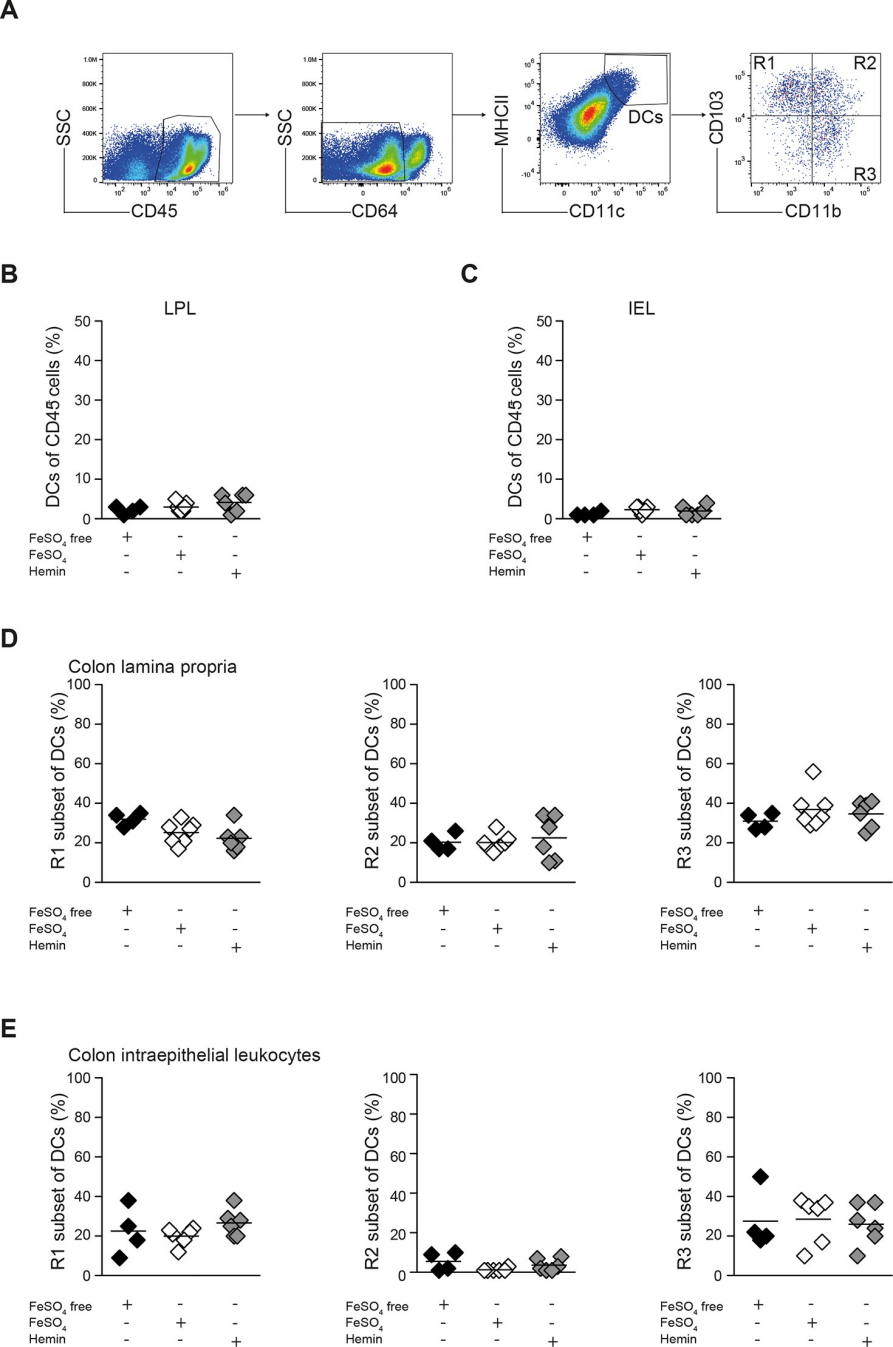
DC subset composition in the colon is not influenced by dietary iron reduction during T cell transfer colitis. *Rag1*^*-/-*^ mice were treated with iron depleted (w/o Fe) or iron supplemented experimental diets (FeSO_4_ or Hemin) and colitis was induced by T cell transfer. Colon LPLs and IELs were prepared on day 17 after T cell transfer and analyzed by flow cytometry. **(A)** Gating strategy for DC subpopulations in colon LPL and IEL fractions. **(B, C)** The frequencies of DCs within viable CD45^+^ leucocytes in colon LPL (b) and IEL (C) are shown. **(D, E)** The frequencies of R1, R2 and R3 subsets within CD64^+^ CD11c^+^ MHCII^high^ DCs within colon LPL (D) and IEL (E) are shown (one of 2 independent experiments with 4–6 animals in one of each dietary group; each dot represents one animal and horizontal lines indicate mean values; one way ANOVA, n.s.).

Besides the macrophages, DCs play an important role in colitis development as inducers of proinflammatory T cell responses [31]. To address if dietary iron content can influence their frequency and phenotype in the colon and their shift from the CD103^+^ to the CD11b^+^ subpopulations during colitis, DC subpopulations in colon LPL and IEL fractions were analyzed by flow cytometry in *Rag1*^*-/-*^ mice in the steady state and during T cell transfer colitis after exposure to different diets. Three distinct DC subsets were distinguished based on CD103 and CD11b expression (Fig 4A). We observed a decrease in the percentage of CD103^+^ CD11b^-^ DCs and an increase in the percentage of CD103^+^ CD11b^+^ DCs after colitis induction (table B in S1 File). However, modulation of dietary iron content had no major effects on the total frequency of DCs or the DC subset composition in the colon during T cell transfer colitis (Fig 4B–4E). Thus, the dietary iron content did not influence DC recruitment to the inflamed colon or DC subset composition during colitis.

### Dietary iron reduction does not influence in vivo T cell differentiation and activation during T cell induced colitis

**Fig 5:**
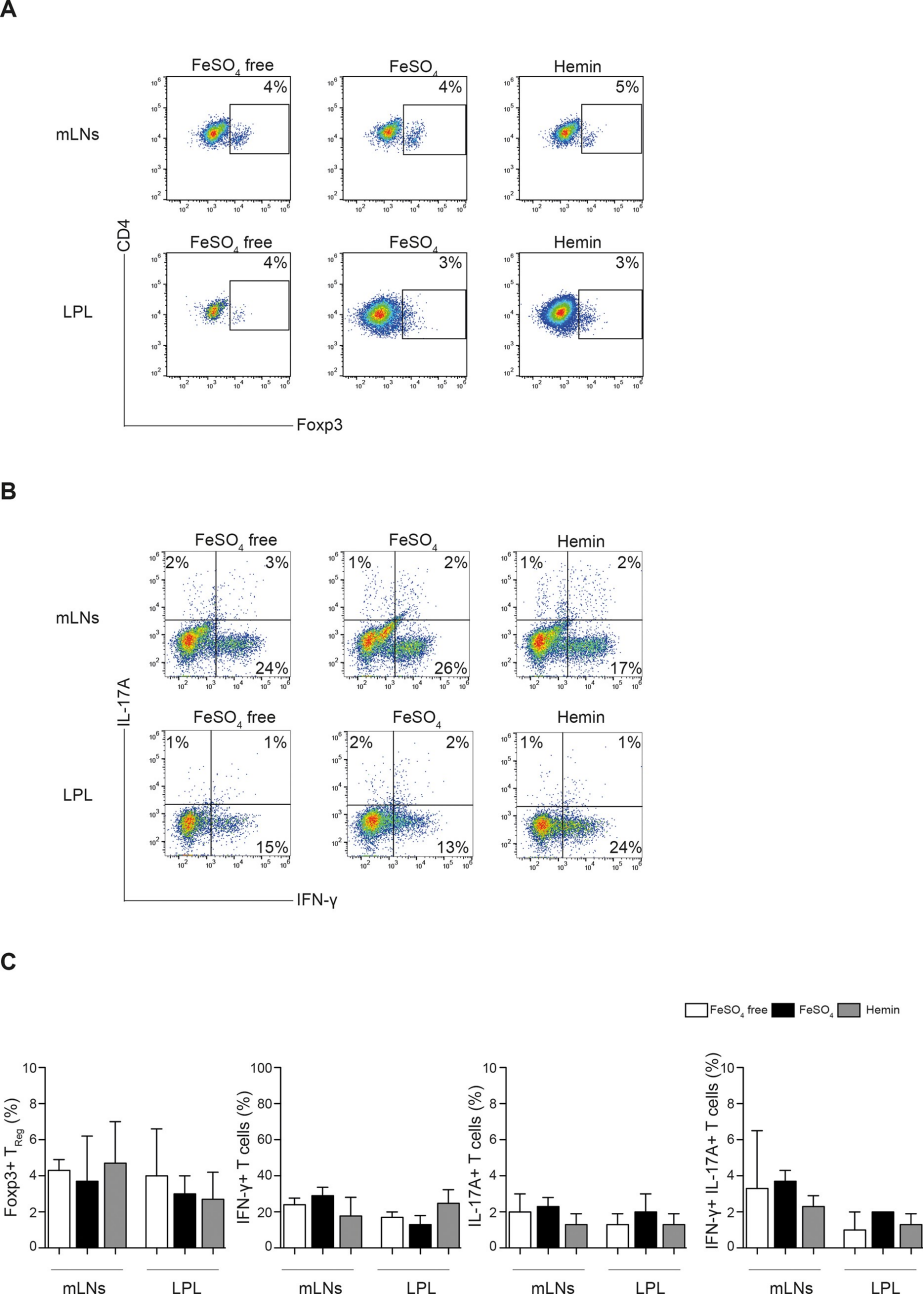
Frequency of Tregs and proinflammatory Th1/Th17 effector cells is not affected by dietary iron content during T cell mediated colitis. *Rag1*^*-/-*^ mice were treated with iron depleted (w/o Fe) or iron supplemented experimental diets (FeSO_4_ or Hemin) and colitis was induced by T cell transfer. MLN cells and colon LPLs were prepared on day 17 after T cell transfer and analyzed by flow cytometry. **(A)** Representative dot plots show Foxp3 expression in CD3^+^ CD4^+^ T cells isolated from mLNs and colon LPL fraction for the 3 experimental groups. Numbers indicate percentages of Foxp3^+^ cells. **(B)** MLN cells and colon LPLs were stimulated with PMA/Ionomycin in the presence of secretion blockers for 6 hours. Representative dot plots show the intracellular IFN-γ and IL-17A content in CD3^+^ CD4^+^ T cells for the respective dietary groups. **(C)** The percentages of Foxp3^+^ Tregs, IFN-γ single positive, IL-17A single positive and IFN-γ/IL-17A double positive Th cells isolated from the indicated organs are shown as mean values ± SD (n = 3 mice per group, one way ANOVA, n.s.).

T cell transfer colitis in lymphopenic mice is driven by CD4+ T cells which get activated, expand and differentiate in response to commensal bacterial antigens and innate immune signals delivered by DCs. Therefore, we explored the effect of luminal iron on CD4^+^ T cell numbers and differentiation in this model. The frequencies of CD4^+^ T cells found in the spleens, mLNs and colon LPL and IEL fractions on day 17 after T cell transfer were comparable between the different dietary treatments (Fig B in S1 File). A small population of Foxp3^+^ Tregs was detected in mLNs and colon LPL as well as spleen and colon IELs with similar frequencies in the 3 dietary groups (Fig 5A and 5C, Fig B in S1 File). The frequencies of CD4^+^ T cells producing IFN-γ^+^, IL-17A^+^ or both cytokines after restimulation in mLNs and colon LPL were also comparable between the groups (Fig 5B and 5C). Thus, in the T cell transfer colitis model dietary iron reduction had no influence on the accumulation of CD4+ T cells in the colon, mLN and spleen and their capacity to produce IFN-γ and IL-17 was not affected. We conclude that iron deprivation in the diet does not protect adult C57BL/6 mice against severe intestinal inflammation in the T cell transfer colitis model and does not change the frequency of relevant immune cell populations in the colon and mLNs.

## Discussion

Clinical observations in IBD patients and experimental studies suggested that iron in the intestinal lumen derived from iron-rich food or iron supplementation exacerbates inflammation in IBD patients [4, 5, 7–12]. This led to the assumption that oral iron replacement therapy for iron-deficiency anemia should be avoided during active IBD and that iron reduction in the diet may be protective. Indeed, evidence for a protective effect of luminal iron depletion was found in a murine model of ileitis [15]. In this model the beneficial effect of luminal iron depletion correlated with reduced ER-stress in the epithelium and changes in the microbiota composition. In contrast, we found that colitis activity in the T cell transfer induced mouse model of colitis was not affected by alterations in the iron content of the experimental diet in recipient mice. Examination of the frequency and activation of relevant innate and adaptive immune cell populations in the colon during colitis revealed that the iron-reduced diet had no significant effect on these parameters of intestinal inflammation.

To investigate the influence of the iron depleted diet on colitis development, we chose to study the CD4^+^ CD62L^+^ T cell transfer induced colitis model. In contrast to chemically induced colitis models in which epithelial disruption is the primary event, T cell transfer colitis is driven by microbiota-dependent proinflammatory Th1/17 cell responses. This is a valid preclinical model, that has been widely used for the development of approved drugs, which are effective in the therapy of Crohn’s disease and ulcerative colitis, such as anti-TNF-α and anti-α4β7-integrin antibodies and probiotics [36, 37, 38] for example. The advantage of this model is that epithelial disruption is not the primary event as in chemically induced colitis models.

As iron cannot be removed from normal chow, the animals were adapted to an experimental semisynthetic diet, which was either iron-free (< 10 mg/kg) or contained 180 mg/kg iron in the form of FeSo4 or hemin–a concentration of iron corresponding to that contained in normal chow [15]. The same semisynthetic diets (with or without FeSo4) have been used in the TNF^ΔARE/WT^ ileitis model where the iron-reduced diet changed the microbiota composition and prevented development of inflammation [15]. In that study it was also shown that the iron-free diet led to depletion of hepatic iron stores but did not cause anemia. This is in line with our results where mice treated with the iron-free diet did not develop overt anemia but showed reduced iron staining in colon tissue sections [15].

Purified diets with different iron concentrations have been tested in chemically induced colitis models in mice and rats where inflammation is initiated by epithelial disruption and massive entry of luminal bacteria into the mucosa of the colon. Seril et al. showed using the DSS colitis model in 8 weeks old mice, that colitis activity and tumor incidence increased with increasing doses of iron (49–490 mg/kg) in the purified diet [12, 39]. Barollo et al. reported increased inflammation and tissue damage in the dinitrobenzene sulfonic acid (DNBS)-induced colitis model in rats fed with a high iron diet (200 mg/kg and 1.7 g/kg) [7]. Furthermore, in several publications, normal chow was supplemented with high doses of iron in different formulations. Carrier et al. observed exacerbation of DSS-induced colitis in rats by addition of 3 and 30 g/kg iron pentacarbonyl iron to the chow correlating with oxidative stress, neutrophil infiltration, NFκB activation and inflammatory cytokine production [8, 16]. Similarly, supplementation with 1% (10 g/kg) carbonyl iron in the chow for 6 weeks started at 4 weeks of age exacerbated colitis and promoted tumor development in the Azoxymethan/DSS mouse model of colitis-associated colon cancer [9]. On the other hand, a recent study using a chronic low dose DSS colitis model in mice demonstrated that a diet with 500 mg/kg ferrous sulfate reduced colitis susceptibility compared to a diet with 50 mg/kg ferrous sulfate [10]. Taken together, most studies that described increased disease severity and tissue damage in chemically induced colitis models supplemented oral iron using much higher doses than those recommended for oral iron replacement therapy in human patients and much higher than the concentrations found in iron-rich foods (e.g. pork liver 180 mg/kg, Canadian Nutrient File, Health Canada, 2015). Our study is the first to investigate the impact of iron reduction in the diet below 10 mg/kg compared to an iron-rich diet with 180 mg/kg ferrous sulfate or hemin in the T cell transfer colitis model and we could not observe a beneficial effect of this intervention. These results contrast with previous observations in the ileitis model in TNF^ΔARE^ mice, where the same semisynthetic iron-depleted diet reduced inflammation compared to the 180mg/kg ferrous sulfate supplemented diet [15].

How can we explain the different results of these two studies? One important aspect is the different pathophysiology of the models which were used. In TNF^ΔARE^ mice intestinal pathology is restricted to the terminal ileum and proximal colon like in human Crohn’s disease. Ileal epithelial cells have a higher basal level of ER-stress and are more responsive to induction of ER-stress *ex vivo* than colon epithelial cells [40]. Therefore the observed effects of dietary iron reduction on ER-stress and intestinal epithelial cell apoptosis may be more pronounced in the ileitis model [15] than in the colitis models.

In TNF^ΔARE^ mice inflammation is driven by dysregulated TNF-α production in response to the microbiota [41, 42] and critically depends on CD8^+^ T cell responses [43]. In contrast, unrestrained microbiota-triggered CD4^+^ Th1/Th17 cells cause inflammation in the T cell transfer colitis model. It cannot be excluded that in the T cell transfer colitis model T cells isolated from mice fed with iron-free diet would have behaved differently after transfer than T cells isolated from mice fed with iron-supplemented diet. Indeed, it was reported that iron can promote expression of GM-CSF and IL-2 in T cells by stabilizing RNA binding protein PCBP1, which promotes stability of Csf2 and Il2 mRNA [44]. However, CD4+ T cells isolated from spleen of TNF^ΔARE/WT^ mice fed with iron-free or iron sulfate supplemented diets did not differ in their ability to produce TNF-α and IFN-γ after TCR stimulation [15].

Another major difference is the kinetic of the inflammatory response. Ileitis develops spontaneously over a period of 8–16 weeks in the heterozygous TNF^ΔARE^ mice, whereas severe colitis develops within 2–3 weeks in the CD4^+^ CD62L^+^ T cell transfer colitis under our experimental conditions. The duration of dietary treatment was comparable between the two studies (8–11 weeks) and allows for full adaptation to the diet as shown previously [15]. Constante et al. reported that even 4 weeks of experimental diet with different concentrations of iron were sufficient to induce changes in the intestinal microbiota composition in adult mic [10]. In that study however microbiota changes did not translate into different susceptibility to colitis induced by low dose DSS. This is in line with the results of a recent clinical trial, which showed that oral iron replacement therapy induces major changes in intestinal bacterial diversity and composition in IBD patients without affecting clinical disease activity [6]. Similarly, an epidemiological study published recently found no significant associations of dietary total iron and heme iron intake with risk of IBD in two large prospective cohorts (from the Nurses Health Study). Only in a subgroup of patients with a specific ulcerative colitis susceptibility locus (a functional coding variant of *Fc𝛾RIIA*) heme iron consumption was significantly associated with risk of ulcerative colitis suggesting that dietary heme iron may promote the development of ulcerative colitis in genetically predisposed individuals but does not have a major effect in the general population [45].

It has been reported that calcium phosphate in the diet can counteract cytotoxic and hyperproliferative effects of luminal hemin [46] and a hemin-supplemented low calcium diet was shown to aggravate chemically induced colitis in rats and mice [47, 48]. However, another study investigated the effect of hemin-supplemented diet with normal calcium content on microbiota and DSS-induced colitis and showed an aggravation of acute DSS colitis by hemin in the diet although the diet was not calcium-depleted [49], which may be due to direct effects of luminal heme iron on the microbiota. In our study dietary iron reduction led to reduced accumulation of iron in the colonic lamina propria and this was prevented by iron supplementation in the form of iron sulfate or hemin. We observed a similar extent of iron staining in colonic lamina propria of mice fed with a hemin-supplemented *vs*. iron-sulfate-supplemented diet with normal calcium content. It is therefore unlikely, but cannot be excluded, that hemin-supplementation did not affect colitis activity in our study is due to the presence of calcium (9450 mg/kg) in the diet.

In our study, we investigated sensitive immunological parameters of inflammation, such as recruitment of monocytes into the colon, changes in DC subset distribution, and activation of Th cells in the colon. Although monocytes/macrophages are equipped to sense luminal iron directly, their response during colitis was not different between mice exposed to iron -reduced diet or diet supplemented with ferrous sulfate or hemin at moderate concentrations corresponding to those found in iron-rich food. Furthermore, accumulation of transferred CD4^+^ T cells in the colon of *Rag1*^*-/-*^ mice and the production of IFN-γ and IL-17 by these cells as well as the frequency of Foxp3^+^ Tregs were unaltered by the iron content of the diet given to recipient mice. These results indicate that in this model which is driven by a microbiota-dependent proinflammatory CD4^+^ T cell response the reduction of dietary iron in the recipient mice did not affect the proinflammatory responses of the transferred T cells. Thus, our results do not provide evidence for a protective effect of dietary iron reduction in this standard mouse model of colitis. It remains to be investigated if such an intervention could be beneficial in a subgroup of ulcerative colitis patients which may be particularly sensitive to luminal iron [45].

## Supporting information

S1 File (PDF)
