# Supplementary material for: Comparison of iron-reduced and iron-supplemented semisynthetic diets in T cell transfer colitis
Source: PLoS One. 2019 Jul 5;14(7):e0218332. doi: 10.1371/journal.pone.0218332 (PMC6611680; doi:10.1371/journal.pone.0218332)
Supplement: S1 File — (PDF) [file pone.0218332.s001.pdf]

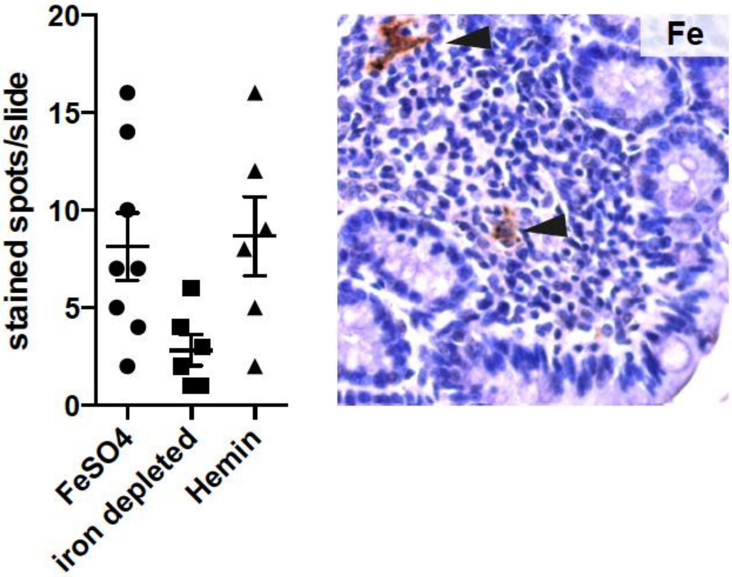

Figure A Detection of iron in colon tissue sections
Left panel: Iron was detected in colon tissue FFPE sections obtained from mice after T cell transfer colitis by DAB-enhanced Prussian blue staining. The number of stained spots per slide was counted. Mean values and SD are shown (pooled results of 2 experiments, n = 8 for FeSo4, n=6 for iron-free and hemin). * $p < 0.025$ (unpaired t-test for FeSO4 vs iron-free and hemin vs iron-free with Bonferroni adjusted significance level). Right panel: Exemplary result of iron staining in colon tissue section, counterstained with hematoxylin. Scale bar: 50 µm.

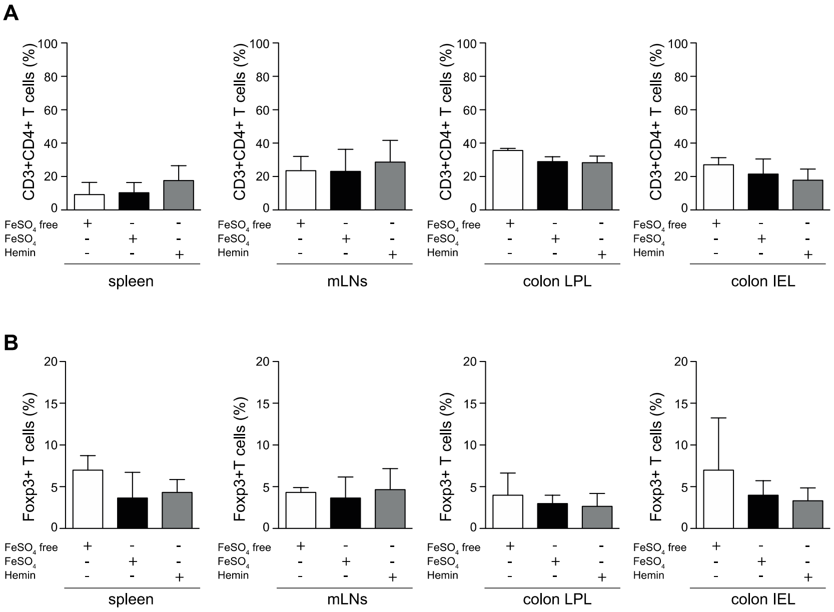

Figure B Frequency of Tregs and proinflammatory Th1/Th17 effector cells during T cell mediated colitis

Rag1$^{-/-}$ mice were treated with iron depleted (w/o Fe) or iron supplemented experimental diets ($FeSO_4$ or Hemin) for 9 weeks and colitis was induced by T cell transfer. Cells were isolated from spleen, MLNs, colon LPLs and colon IELs on day 17 after T cell transfer and analyzed by flow cytometry. The percentages of CD4+ T cells (A) and the percentages of Foxp3$^+$ Tregs within CD4+ T cells (B) were determined (mean values ± SD, n= 3 mice per group, one way ANOVA, n.s.).

Table A*

Diet		WBC (10^9/l)	RBC (10^{12}/l)	Hb (g/l)	HCT (%)	MCV (fl)*	MCH (pg)
$FeSO_4$ -free	Before colitis	2.6 ± 1.4	7.6 ± 3.0	14.9 ± 3.9	43.6 ± 9.1	53.3 ± 1.1	14.4 ± 2.5
	During colitis	11.7 ± 3.0	10.9 ± 1.3	19.4 ± 2.6	64.7 ± 11.1	43.87 ± 2.1	14.3 ± 1.7
$FeSO_4$	Before colitis	2.3 ± 1.8	7.2 ± 4.1	16.4 ± 3.6	40.9 ± 7.6	50.7 ± 2.0	14.8 ± 1.3
	During colitis	10.1 ± 2.9	12.8 ± 2.9	22.2 ± 2.5	69.5 ± 10.6	47.9 ± 1.6	14.8 ± 0.9
Hemin	Before colitis	4.3 ± 3.2	6.7 ± 2.9	15.8 ± 3.4	44.6 ± 8.1	52.5 ± 1.6	13.9 ± 2.2
	During colitis	9.6 ± 3.8	9.9 ± 1.1	21.5 ± 2.9	61.1 ± 6.3	44.6 ± 2.4	15.4 ± 1.2

***Hematological parameters before and after colitis induction**

Rag1$^{-/-}$ mice were treated with three different experimental diets (iron depleted (w/o Fe) or iron supplemented ($FeSO_4$ or Hemin) diet) for 9 weeks, colitis was induced by T cell transfer. Blood samples were analysed after 9 weeks of diet before T cell transfer and on day 17 after T cell transfer. White blood cell count (WBC); red blood cell count (RBC), hemoglobin (Hb), hematocrit (HCT), mean corpuscular volume (MCV) and mean corpuscular hemoglobin (MCH) were analysed. Mean and SD are shown (n=7 for $FeSO_4$; n=6 for w/o Fe; n=6 for Hemin). One-way analysis of variance (ANOVA) was performed to compare differences between dietary groups. * indicates $p < 0.05$. Only MCV was significantly different between the dietary groups both before and during colitis.

Table B*

Diet		CD103$^+$CD11b$^-$ of DCs (%)	CD103$^+$CD11b$^+$ of DCs (%)	CD103$^-$CD11b$^+$ of DCs (%)
$FeSO_4$-free	Steady state	38.8 ± 5.1	11.2 ± 3.8	16.6 ± 5.0
	Colitis	32.0 ± 3.2	20.3 ± 4.3*	31.0 ± 4.1*
$FeSO_4$	Steady state	42.2 ± 9.0	4.8 ± 3.1	19.2 ± 7.8
	Colitis	25.1 ± 5.6*	20.1 ± 4.1*	36.9 ± 9.3*
Hemin	Steady state	33.3 ± 9.0	8.5 ± 2.4	33.3 ± 6.2#
	Colitis	22.3 ± 6.3	22.5 ± 11.0*	34.6 ± 6.6

***Frequency of DC subpopulations in colon LPL in steady state vs. colitis** Cells were isolated from colon LPL and DC subpopulation frequency analyzed by flow cytometry after dietary treatment (steady state) or on day 17 after T cell transfer into Rag1-/- mice treated with the indicated diets (colitis). The percentages of indicated DC subpopulation within the DC gate are shown (mean ± SD). * indicate significant differences between colitis and steady state (unpaired t test, $p < 0.05$, n = 4-6 mice per group). # indicates that mice receiving hemin-supplemented diet had a significantly higher percentage of CD103- CD11b+ cDCs in the steady state compared to the two other diet groups (2-way ANOVA, Tukey's multiple comparison test, $p < 0.05$).

Supplementary Materials and Methods

Hematological analysis

Blood samples were collected from either facial vein or postmortem by cardiac puncture, depending on the time point of the sampling in the feeding experiments. For facial blood collection, mice were pinched behind the jawbone using a 5 mm lancet and blood was collected into EDTA containing collection tubes. Cardiac puncture was performed for terminal blood collection. Mice were sacrificed by CO_2 asphyxia. The blood was drawn from the heart using a 0.5 ml insulin syringe and transferred into EDTA containing collecting tubes. Blood samples were diluted in phosphate-buffered saline (1:3). White blood cell count (WBC), haemoglobin (Hb), haematocrit (HCT), mean corpuscular volume (MCV) and mean corpuscular haemoglobin (MCH) were measured by the Clinical Chemistry department using Sysmex XT-2000i-1 Hematology Analyzer (Sysmex Europe GmbH, Norderstadt, Germany).

Iron staining in tissue sections

Ferric iron staining in colon tissue FFPE sections was performed according to Meguro et al. (1) using the Iron Stain Kit (Sigma Aldrich) for Prussian Blue staining and the DAB-Substrate Kit (Vector Laboratories, Burlingame, CA, USA) for enhancement. Counterstaining was performed with Hematoxylin (Merck Millipore, Burlington, MA, USA).

Reference

1. Meguro R, Asano Y, Odagiri S, Li C, Iwatsuki H, Shoumura K. Nonheme-iron histochemistry for light and electron microscopy: a historical, theoretical and technical review. Archives of histology and cytology. 2007;70(1):1-19.
